# A sporadic pediatric case of a spinal dumbbell-shaped epithelioid malignant peripheral nerve sheath tumor with a novel germline mutation in SMARCB1: a case report and review of the literature

**DOI:** 10.3389/fneur.2023.1178651

**Published:** 2023-05-25

**Authors:** Maoyang Qi, Nan Jiang, Wanru Duan, Zan Chen

**Affiliations:** ^1^Department of Neurosurgery, Xuanwu Hospital, Capital Medical University, Beijing, China; ^2^Spine Center, China International Neuroscience Institute (CHINA-INI), Beijing, China; ^3^Lab of Spinal Cord Injury and Functional Reconstruction, Xuanwu Hospital, Capital Medical University, Beijing, China

**Keywords:** epithelioid malignant peripheral nerve sheath tumor, SMARCB1/INI-1 mutation, spinal dumbbell-shaped tumor, second-hit loss, radical resection

## Abstract

Malignant peripheral nerve sheath tumors (MPNSTs) are commonly associated with poor prognosis and are primarily caused by germline mutations in the SMARCB1/INI-1 gene. However, these tumors are rarely found in the spine. This case report presents the case of a 3-year-old boy diagnosed with a lumbosacral dumbbell-shaped epithelioid MPNST, an extremely uncommon manifestation. Immunohistochemistry revealed the complete absence of the SMARCB1/INI-1 protein, and genetic testing identified a novel germline mutation in the SMARCB1/INI-1 gene in both the patient and his father, suggesting a “second-hit loss.” One year of follow-up after the tumor's radical resection revealed no suspected metastasis. This case report offers novel genetic research results regarding spinal dumbbell-shaped MPNSTs. Six studies, including 13 cases associated with spinal dumbbell MPNST, were included in the literature. The range of age of these patients varied from 2 to 71 years. Of the 12 known patients diagnosed with spinal dumbbell MPNST, only one received radiation therapy, while the rest underwent surgery. Two patients who underwent partial resection had metastases after surgery, while one of the five patients who underwent complete surgical resection alone had no distant metastases and a good prognosis, indicating that radical resection is more likely to be effective in inhibiting distant metastasis and improving the prognosis.

## 1. Introduction

Epithelioid malignant peripheral nerve sheath tumors (EMPNSTs) are a highly malignant and extremely rare variant of malignant peripheral nerve sheath tumors (MPNSTs), accounting for only 5% of all MPNST variants (1–4). Primary EMPNSTs, particularly paravertebral dumbbell-shaped tumors, are rarely observed in the spine. These tumors mainly affect individuals aged 20 to 50 years, and they are uncommon in women. EMPNSTs occur in superficial and deep soft tissues, similar to conventional MPNSTs. However, studies have shown that EMPNSTs are usually smaller and more superficial than conventional MPNSTs. EMPNSTs can occasionally arise from schwannomas, while MPNSTs are closely associated with neurofibromatosis type 1 (NF1) and occasionally arise from epithelioid schwannomas or neurofibromas (5, 6). EMPNSTs are characterized by homogeneous epithelioid tumor cells exhibiting a multilobular growth pattern with myxoid and fibrous stroma-associated nests and cords. EMPNSTs are identified by diffuse and strong staining of the S100 protein, and immunohistochemistry can show the absence of SMARCB1/INI-1 protein expression in 50% to 67% of EMPNSTs (7).

The study of EMPNSTs has been significantly enhanced by advances in gene-sequencing technology. In this case report, we present the genetic research findings of a spinal dumbbell-shaped EMPNST and provide new insights into the surgical strategy and exploration of EMPNSTs in children. Furthermore, we reviewed all reported spinal dumbbell-shaped EMPNSTs and evaluated the effectiveness of radical resection for recurrence and metastasis.

## 2. Case presentation and results

**Figure 1:**
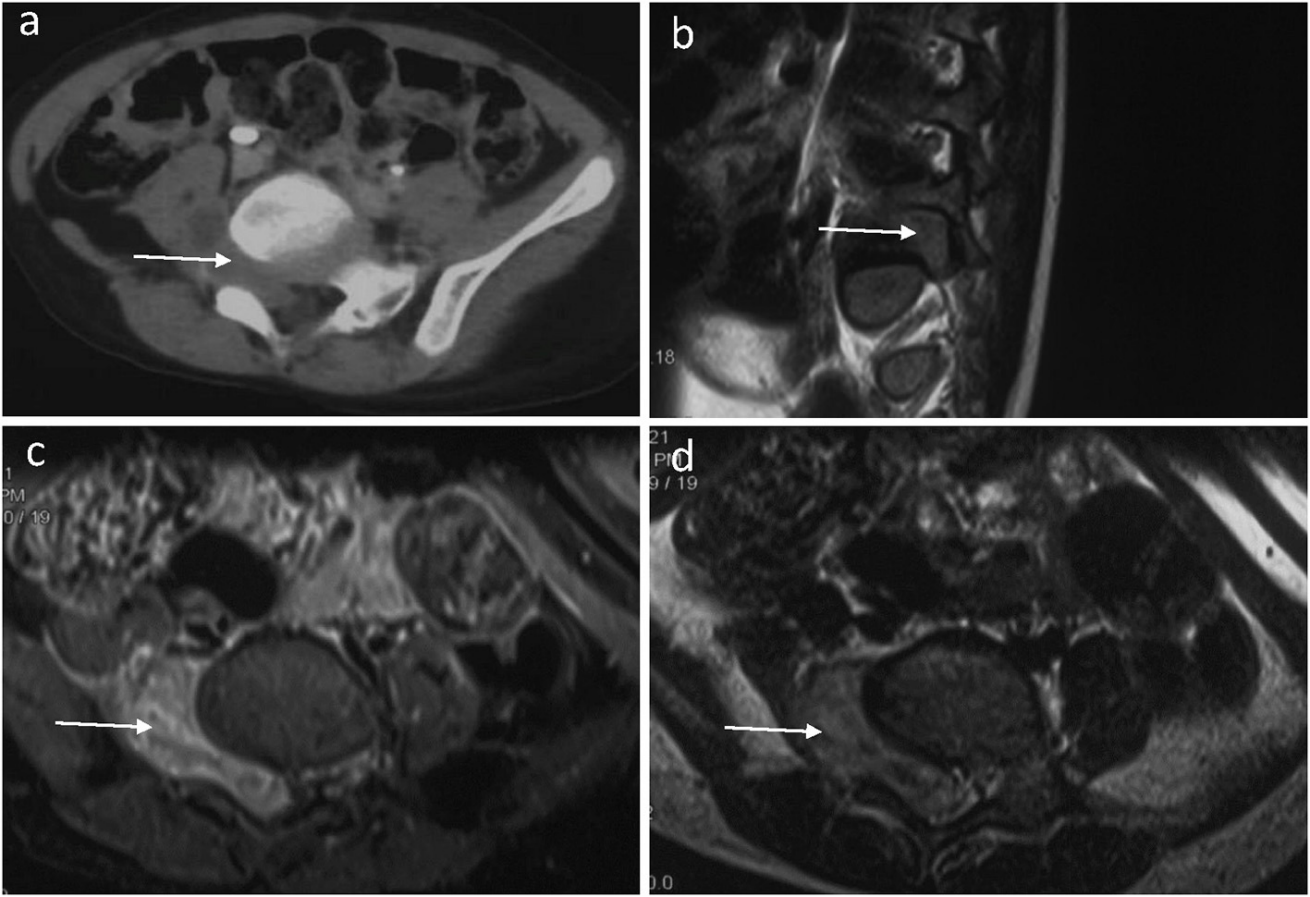
A preoperative horizontal CT image of the lumbar spine **(a)** showed an abnormal density shadow (arrow) in the L5/S1 segment. A sagittal T2-weighted fat-suppressed MR image of the lumbar spine **(b)** showed that the right intervertebral foramen of the L5 segment is filled by tumor tissue (arrow) and the compressed nerve root. A horizontal T2-weighted fat-suppressed MR image of the lumbar spine **(c)** showed that the dumbbell-shaped tumor has an equal or slightly high signal (arrow) in the L5/S1 segment. The spinal cord deviated to the left, and the right foramen of L5/S1 widened. A horizontal T1-weighted fat-suppressed MR image **(d)** showed a slightly hypointense (arrow) tumor in the L5/S1 segment.

A 3-year-old boy presented with a 3-month history of limping and intermittent pain in the right plantar. He became unwell and was reluctant to walk for 2 months before being admitted to the hospital. Pelvic computed tomography (CT) scanning revealed a mass with a heterogeneous signal arising through the foramina at the right-sided Lumbar 5 (L5) and Sacral 1 (S1) (Figure 1a). T2-weighted magnetic resonance imaging (MRI) showed a dumbbell-shaped mass filling the foramina with mixed areas of isointense and slightly hyperintense signals compared to skeletal muscle (Figures 1b–d). The mass measured 4.2 x 3.0 x 2.1 cm (anteroposterior, transverse, and craniocaudal dimensions). Further physical examination revealed decreased muscle strength and atrophy of the right lower limb.

**Figure 2:**
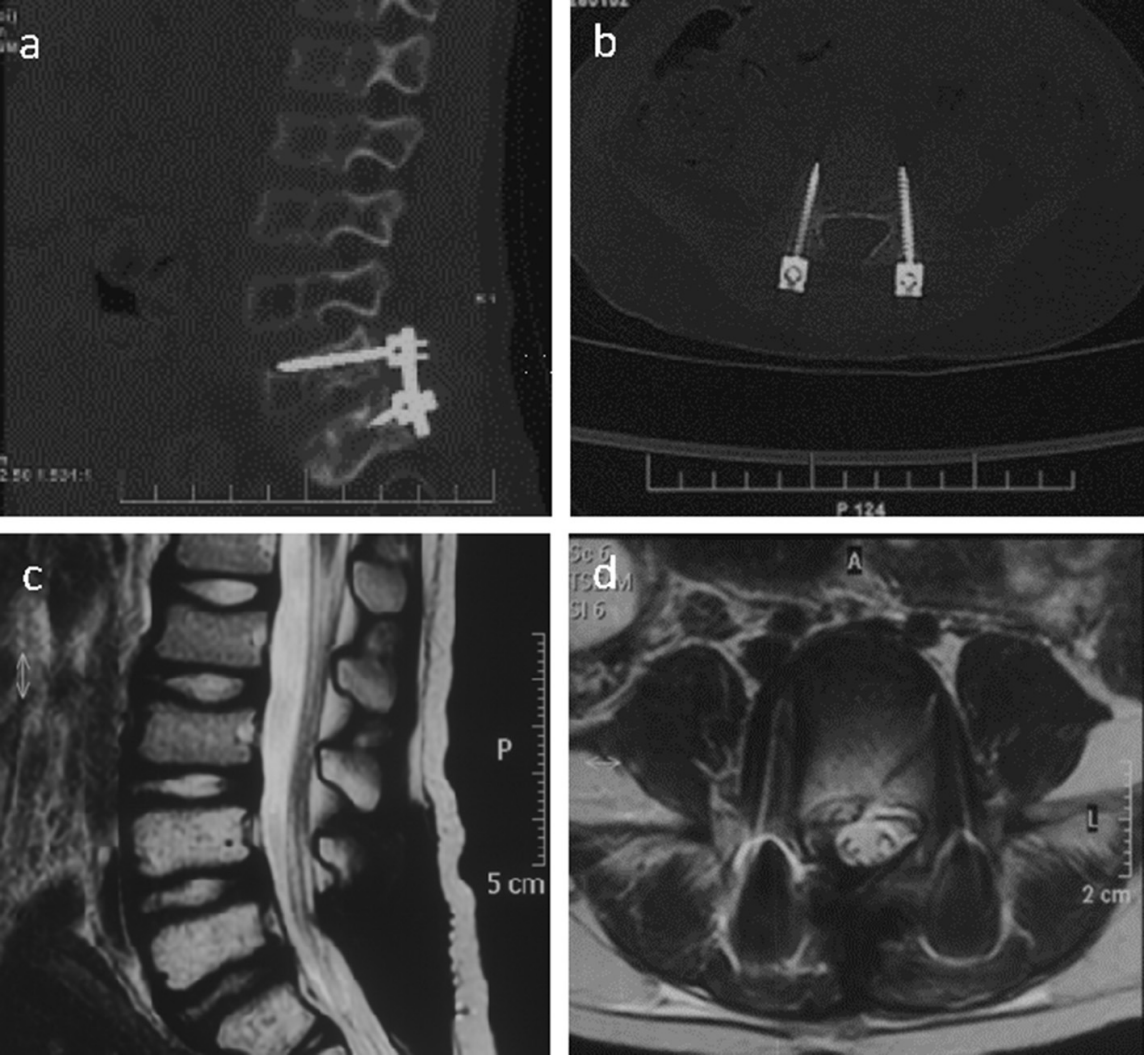
Right sagittal CT scan of the lumbar spine image **(a)** showed the placement of pedicle screws at the L5/S1 segment and the fixation with titanium rods 1 week after the operation. The CT scan of the L5 segment image **(b)** showed that the pedicle screws are well positioned on both sides. The sagittal T2-weighted image of the lumbar spine MR **(c)** was normal 1 year after the operation, and the horizontal image showed a normal signal in the L5 intervertebral foramen **(d)**.

Radical tumor resection with the microscope was performed, and bilateral pedicle screws were implanted at L5 and S1. The patient's muscle strength improved after the operation, and he could walk independently. Systemic postoperative chemotherapy (vincristine + cyclophosphamide + doxorubicin/ifosfamide + etoposide) and 160 cGy single pelvic irradiation were administered for 28 days. Follow-up CT (Figures 2a, b) and MRI (Figures 2c, d) findings 1 week and 1 year after surgery showed no evident recurrence of the tumor.

**Figure 3:**
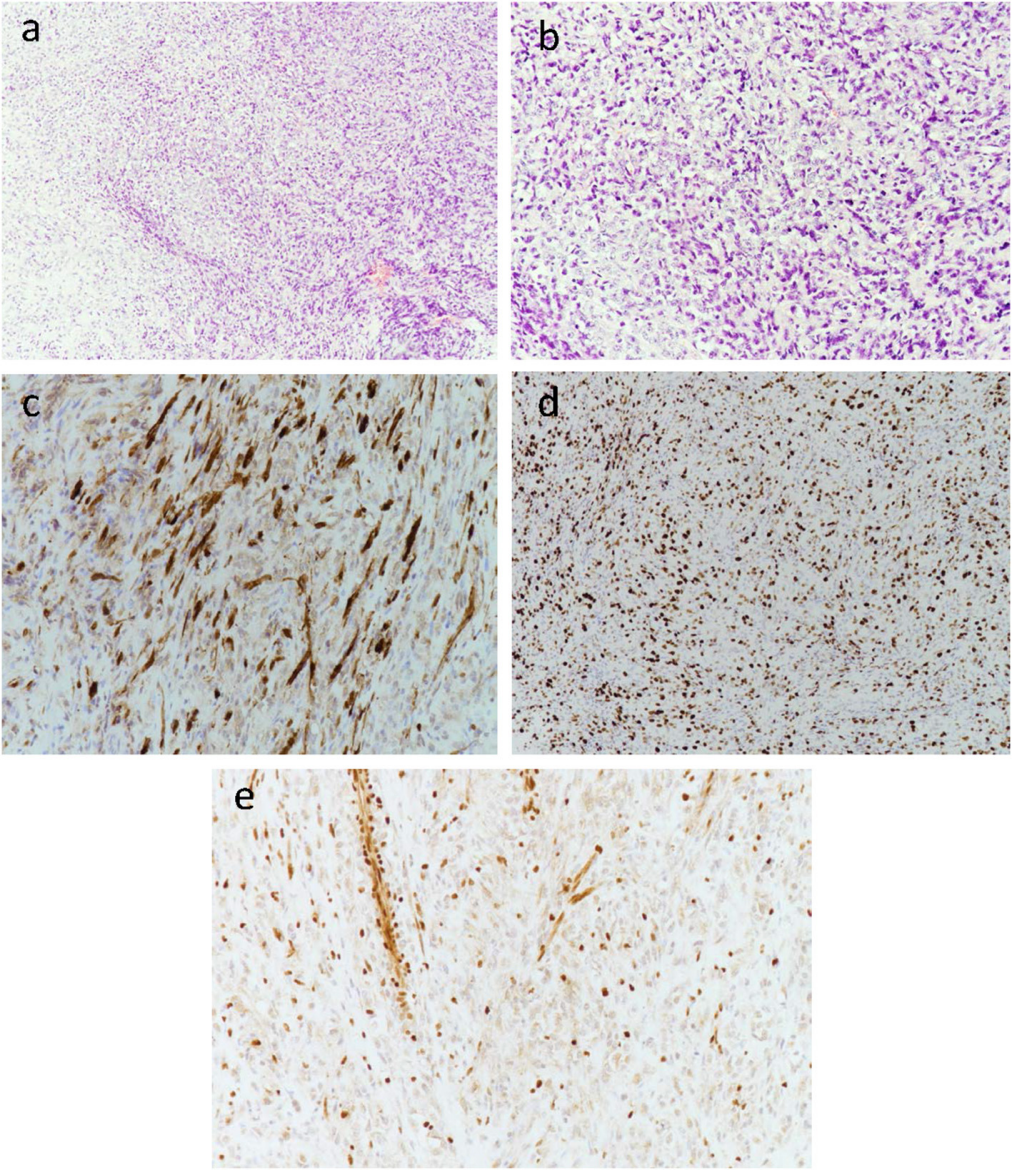
Low-power view of diffuse growth of spindle tumor cells **(a)**. Medium-power view of some tumor cells with clear cytoplasm and an epithelioid structure. Most of the specimens are obvious malignant tumors with active focal growth as well as mitotic images **(b)**. The tumor was sporadically positive for the S100 protein by immunohistochemistry **(c)**. Immunohistochemistry for Ki-67 was performed **(d)**. Immunohistochemical staining showed the complete absence of SMARCB1/INI-1 **(e)**, with retained expression in normal endothelial cells.

Immunohistochemistry revealed diffuse growth of spindle cells and polygonal cells visible in the low-power view (Figure 3a). Under the medium-power view, some tumor cells showed clear cytoplasm and an epithelioid structure. Most of the specimens were malignant tumors with active focal growth and mitotic images (Figure 3b). The tumor was sporadically positive for the S100 protein by immunohistochemistry (Figure 3c). Immunohistochemistry for Ki-67 was performed (Figure 3d), and immunohistochemical staining showed complete deletion of SMARCB1/INI-1 (Figure 3e).

Blood samples were collected from the patient and his parents, and gene sequencing was performed (IWES, Cipher Gene, China). Germline copy number loss of heterozygosity in the q11.21-q11.23 region of chromosome 22 (~3.018 Mb) was found in the patient, which was inherited from his father. Panel sequencing (GeneseeqPrime™, Geneseeq, Nanjing, China) was performed on the tumor after the operation. A frameshift mutation was detected [c.606del (p.D202Efs^*^7)] in exon 5 of the SMARCB1 gene, which was considered a “second-hit loss” (ADDL Figure 1).

We also searched the key terms “malignant peripheral nerve sheath tumor,” “dumbbell,” and “spinal” in PubMed and the Web of Science databases. Finally, only eight studies associated with spinal dumbbell MPNST were included in the literature. Six of the eight articles contained detailed reports of individual cases, and the remaining two were case series that could not be included in the table because of their incomplete information. Michael M. Safaee reported on five patients with MPNST who underwent radical tumor resection; the complication rate was 20%. One of them did not have a dumbbell-shaped MPNST, so we did not discuss it in detail (8). Matsumoto Yoshihiro reported on another 11 cases of spinal dumbbell-shaped MPNST, which were described morphologically only, so we also did not include them in the table (9).

All included studies were spinal dumbbell-shaped MPNSTs, and the mean follow-up ranged from 1 month to 120 months. The age, sex, pathology type, lesion node, treatment, and prognosis are summarized in Table 1. However, many studies did not report adverse events. The range of age of these patients varied from 2 to 71 years. A total of 13 cases had 11 dumbbell-shaped MPNSTs and two dumbbell-shaped EMPNSTs. Of all the cases, four cases occurred in the cervical spine, two in the thoracic spine, three in the lumbar spine, one in the lumbosacral spine, two in the sacral spine, and one in an unknown location.

**Table 1:** The summary of cases and their clinical outcomes in spinal dumbbell-shaped EMPNST.

**References**	**Age**	**Sex**	**Type**	**Spinal cord level**	**Treatment**	**Follow-up period (mos)**	**Metastasis**	**Outcome**
Sasanori et al. (10)	25	Male	MPNST	Cervical	Incomplete resection	50	Yes	Faver
Pan et al. (11)	14	Female	MPNST	Lumbosacral	OP	1	NO	Faver
Dawes et al. (12)	44	Male	MPNST	Cervical	Incomplete resection	2	Yes	poor
Matsumoto et al. (13)	42	Male	MPNST	Cervical	OP and RT	10	NA	poor
	71	Female	MPNST	Lumbar	RT	22	NA	Faver
	21	Male	MPNST	Lumbar	OP	120	NA	Poor
	70	Male	MPNST	Thoracic	OP	5	NA	Poor
	2	Female	MPNST	Thoracic	OP	88	NA	Poor
	37	Male	MPNST	Sacrum	OP and RT	8	NA	Poor
	69	Female	MPNST	Cervical	OP	84	NA	Poor
	35	Male	MPNST	Lumbar	OP, CT, and RT	13	Yes	Poor
Schaefer and Hornick, (14)	43	Male	EMPNST	NA	NA	NA	Yes	NA
Jiwani et al. (15)	54	Female	EMPNST	Sacrum	Incomplete resection	NA	NA	Faver

NA, not application; OP, operation; RT, radiotherapy; CT, chemotherapy.

Of the 12 known patients diagnosed with spinal dumbbell MPNST, all but one were treated surgically with single-dose radiotherapy. Two patients who underwent partial resection had metastases after surgery, while one of the five patients who underwent complete surgical resection alone had no distant metastases and a good prognosis, indicating that radical resection is more likely to be effective in inhibiting distant metastasis and improving the prognosis. Although these preliminary findings are promising, a larger sample size is urgently required to confirm our findings.

## 3. Discussion

Malignant peripheral nerve sheath tumors (MPNSTs) are rare and high-grade neurogenic tumors with an incidence of approximately 0.001%, accounting for 3%–10% of malignant soft tissue neoplasms. When the spine is involved, a dumbbell-shaped mass with bone erosion can be observed. MPNSTs seldom arise in the vertebral body. A variant of MPNSTs called epithelioid MPNSTs (EMPNSTs) accounts for approximately 5% of all MPNSTs cases. EMPNSTs occur in other organs, such as superficial and deep soft tissue, and have been previously reported in the kidney, bladder, ovary, trachea, lung, ileum, prostate, pleura, mediastinum, and retroperitoneum (16). The pathologic diagnosis for this spinal dumbbell-shaped tumor was EMPNST, which is extremely rare based on prior reports.

In general, sarcomas arising from schwannomas often exhibit an epithelioid morphology, including epithelioid malignant peripheral schwannomas and epithelioid angiosarcomas, whereas most neurofibromas cause conventional spindle cell EMPNSTs (2, 17). T1-weighted MRI of EMPNSTs shows a slightly hypointense signal, and an isointense or slightly high mixed signal can be observed on T2-weighted MRI. The enhanced MRI shows a noticeable heterogeneous signal. Genetic sequencing of the family revealed a heterozygous loss of germline copy number in the q11.21–q11.23 region of chromosome 22 (~3.018 Mb), which was inherited from the patient's father. Panel sequencing of the tumor revealed a frameshift mutation in exon 5 of SMARCB1. To our knowledge, this is the first report of this novel mutation site in the SMARCB1 gene in EMPNSTs. Carter et al. reported on a case of EMPNST arising from a schwannoma with a germline mutation of SMARCB1, specifically a two-base-pair insertion in exon 3 of the SMARCB1 gene (c.245_246insAT).

Loss of SMARCB1/INI-1 copy number is widespread in more than 90% of malignant rhabdoid tumors and epithelioid sarcomas (18, 19), such as malignant peripheral nerve sheath tumors, renal rhabdoid tumors, atypical teratoid rhabdoid tumors (ATRT) in the central nervous system tumors, and extrarenal pediatric rhabdoid tumors (20). It has been reported that approximately 25% of patients with rhabdoid tumors (RTs) have SMARCB1 germline alteration, which leads to rhabdoid tumors or predisposition syndrome type 1 (RTPS1) (21). Conventional MPNST usually arises from benign neurofibromas, characterized by inactivation of NF1, CDKN2A, SUZ12, or EED, leading to loss of chromatin mark trimethylation at lysine 27 of histone 3 (H3K27me3) (22, 23). However, similar to conventional MPNST, the inactivation of SMARCB1 and CDKN2A are the two most common genetic variants in EMPNST (23). Loss of SMARCB1/INI-1 copy number has been reported in 50%−67% of EMPNSTs, which has been reported to be associated with the Hedgehog pathway (24). In previous studies, loss of SMARCB1 tumor suppressor function may be critical for the progression of tumors, with mutational inactivation occurring either as mononucleotide mutations or as localized homozygous or hemizygous deletions throughout the coding region of the gene. SMARCB1 might be downregulated at the epigenetic level (25). Inga-Marie Schaefer at el. reported that different driver events were detected in SMARCB1-wild-type EMPNST, which is associated with tumor suppressors in MPNST development and progression. However, the genetic mechanisms that promote the transformation of epithelioid forms into malignant tumors remain unknown.

Interestingly, they found no significant morphological differences in EMPNST with or without combined SMARCB1 mutations (23). Schaefer et al. reported that the proportion of SMARCB1 gene mutations in EMPNSTs reached 81%, indicating that SMARCB1 is a key factor in the oncogenic driver. The protein encoded by the SMARCB1 gene is the core subunit of SWI/SNF, which is an ATP-dependent chromatin regulatory complex that can suppress tumors (14). Tumor-related SMARCB1 mutations are often attributed to biallelic inactivation. The “first hit” is a germline mutation in the SMARCB1 allele, followed by a “second hit” of somatic cells. This theory was confirmed by the family genetic sequencing result and the panel sequencing of postoperative tumor tissue.

The pathological differential diagnosis of EMPNSTs includes malignant melanoma, myoepithelial carcinoma, proximal epithelioid sarcoma, epithelioid myxofibrosarcoma, and extraosseous myxoid chondrosarcoma (EMCS). EMPNSTs are characterized by nodules, oval cells, and cords with prominent nucleoli and usually manifest a focal striated morphology or clear cytoplasm. Unlike conventional MPNSTs, EMPNSTs typically show diffuse and strong staining of the S100 protein. The loss of expression of the melanoma-associated antigens can be observed, such as Melan-A/Mart-1, HMB-45, and MiTF, which can differentiate melanoma.

Currently, the mainstay of treatment for EMPNSTs involves radical surgical resection combined with postoperative chemoradiotherapy and neoadjuvant therapy (26). From the 13 clinical data points, it is difficult to judge the prognosis of surgical treatment, but radical resection is more likely to be effective in inhibiting distant metastasis and improving the prognosis (Table 1) (10–13, 15, 23, 27). While EZH2 inhibitors have been reported to control the progression of EMPNSTs, recurrence remains (14). More cooperation and efforts are needed to explore potential treatments for EMPNSTs.

We report a case of a child diagnosed with paravertebral EMPNST who was confirmed to have inherited the germline SMARCB1 mutation from his father. The genetic evaluation of the tumor revealed a novel frameshift mutation in exon 5 of SMARCB1. Surgical radical resection combined with postoperative radiotherapy and chemotherapy suppressed the progression of the tumor. A literature review also highlighted that radical resection may be necessary to achieve local control to avoid recurrence.

## Data availability statement

The datasets presented in this article are not readily available because of ethical and privacy restrictions. Requests to access the datasets should be directed to the corresponding authors.

## Ethics statement

Ethical review and approval was not required for the study on human participants in accordance with the local legislation and institutional requirements. Written informed consent to participate in this study was provided by the participants' legal guardian/next of kin. Written informed consent was obtained from the individual(s), and minor(s)' legal guardian/next of kin, for the publication of any potentially identifiable images or data included in this article.

## Author contributions

MQ participated in all aspects of this report, including patient management, and draft writing. NJ managed the patient and reviewed the manuscript. WD and ZC managed the patient and supervised the study. All authors have read and approved the final manuscript and agree to be held accountable for all aspects of the report.

## Supplementary material

Supplementary Figure 1 The panel sequencing of the tumor revealed a frameshift mutation [c.606del (p.D202Efs^*^7)] in exon 5 of the SMARCB1 gene, demonstrating the deletion of the base C.
