# Supplementary figures and images for: A sporadic pediatric case of a spinal dumbbell-shaped epithelioid malignant peripheral nerve sheath tumor with a novel germline mutation in SMARCB1: a case report and review of the literature
Source: Front Neurol. 2023 May 25;14:1178651. doi: 10.3389/fneur.2023.1178651 (PMC10248439; doi:10.3389/fneur.2023.1178651)

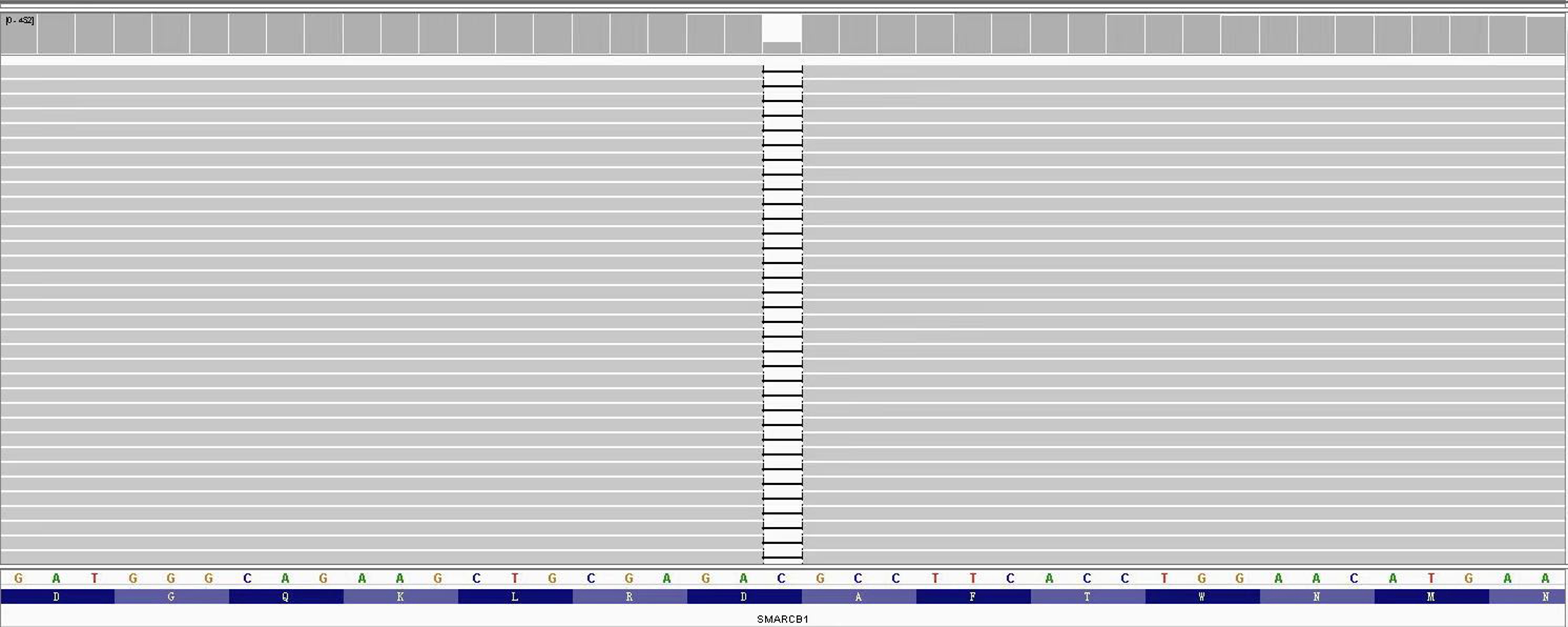

Supplement: Supplementary Figure 1 — The panel sequencing of the tumor revealed a frameshift mutation [c.606del (p.D202Efs*7)] in exon 5 of the SMARCB1 gene, demonstrating the deletion of the base C. [file Image_1.JPEG]
